# A Real-World Clinical Trial Evaluating Satisfaction and Experiences with a Dexamethasone Mouth Film Compared to Corticosteroid Tablets as Rescue Medication for Moderate to Severe Acute Allergic Reactions

**DOI:** 10.3390/jmahp14030043

**Published:** 2026-07-27

**Authors:** Leif Bjermer, Göran Tornling, James Kereki, Karin Wahlberg, Bahram Javizian, Jonas Hjelmgren

**Affiliations:** 1 Department of Respiratory Medicine and Allergology, Lund University, SE-221 85 Lund, Sweden; 2 Division of Immunology and Respiratory Medicine, Department of Medicine Solna, Karolinska Institutet, SE-171 76 Stockholm, Sweden; 3 Acucort AB, SE-223 81 Lund, Sweden; 4 The Swedish Institute for Health Economics, SE-223 61 Lund, Sweden; 5 Kåbohälsan, SE-752 37 Uppsala, Sweden

**Keywords:** acute allergic reactions, corticosteroids, dexamethasone, betamethasone, mouth film

## Abstract

Despite the need for immediate treatment during acute allergic reactions (AARs), many patients do not consistently carry medication. A new mouth-dissolving dexamethasone film offers a portable, easily administered alternative to corticosteroid tablets. This non-randomized, open-label, low-interventional real-world trial assessed satisfaction (accessibility and safety/security) with the mouth film versus betamethasone tablets in adults prescribed tablets for moderate to severe AARs at a Swedish primary care center. Over six months, participants had access to both treatments and reported monthly via an electronic diary; qualitative responses were converted to Likert scales where applicable. Of 50 enrolled, 44 provided diary data (mean age 50 years, 74% female, 58% had an epinephrine autoinjector). In total, 189 responses were collected: 98% stated that the participant was satisfied or very satisfied with mouth film accessibility versus 58% for tablets (mean Likert scores 3.5–3.7 vs. 2.5–2.8; *p* < 0.001). A higher feeling of safety with the mouth film versus tablets was reported in 78% of responses and improved medication carriage compliance in 77%. At six months, 69% preferred the film. Of 18 AARs in total reported by 13 participants, the mouth film was chosen for treatment of 16 (11 participants); in 94%, the mouth film was found immediately accessible, and in 94%, the efficacy was rated as good or very good. In conclusion, the mouth film improved perceived accessibility, safety/security, and medication-carriage compliance compared with tablets for the treatment of AARs.

## 1. Introduction

Allergic conditions affect around 10–30% of the global population and pose a growing global public health concern [1]. Some types of allergies can manifest as acute allergic reactions (AARs) characterized by sudden, exaggerated immune responses commonly triggered by allergens in for instance foods, insect venoms, animal fur, pollen and medications [2]. Food allergies, one of the leading causes of AARs [3], are becoming increasingly common with prevalence estimates exceeding 10% in some high-income countries [4].

AARs represent a broad spectrum of symptom severity ranging from mild skin irritation to moderate or severe symptoms, such as urticaria (hives), angioedema (swelling of the face, lips, or throat), respiratory distress, gastrointestinal discomfort, and cardiovascular symptoms [5]. The symptoms typically begin within minutes to hours of exposure to allergens and can escalate rapidly in severity, especially in anaphylaxis, the most severe form of AARs where multiple organ systems are involved with potentially fatal consequences [6]. The severity of AARs is on a continuum and the distinction between a severe AAR and anaphylaxis is not always clear [5,6].

Short-term treatment of AARs is guided by the severity of symptoms [2]. For mild symptoms, such as localized itching or hives, oral antihistamines are generally sufficient, whereas moderate to severe non-anaphylactic AARs are typically managed by antihistamines in combination with corticosteroids (i.e., betamethasone, dexamethasone or prednisolone) [7]. Anaphylaxis requires immediate intramuscular administration of epinephrine, which can be self-administered using an epinephrine autoinjector (EAI). Corticosteroids in combination with bronchodilators and/or antihistamines are often used as second-line treatment in anaphylaxis [3,5].

The unpredictable nature of AARs, both in terms of timing and severity, can lead to anxiety and avoidance behaviors, and may compromise quality of life [8,9,10,11]. Anxiety may be augmented by concerns related to disease management and medication access in an acute situation. Although immediate treatment of AARs is crucial to avoid the development of severe symptoms, studies have reported that a relatively large proportion of individuals with AARs fail to always bring their medication with them [9,12,13]. Furthermore, a tablet formulation needs access to water, and difficulties in swallowing oral medication have been reported among individuals with AARs [9]. This indicates a need for interventions that can facilitate optimal disease management among individuals with risk for AARs.

Fast-dissolving oral films are an innovative drug-delivery system that dissolves or disintegrates quickly in the mouth and releases the active pharmaceutical ingredient without the need for additional water. This system offers several advantages, including ease of administration, rapid onset of treatment, and improved patient compliance [14]. A newly developed dexamethasone film provides an alternative mode of corticosteroid administration for the management of AARs [15]. The film is small and easily portable (fits into a mobile case), eliminating the need to carry both conventional tablets and water for administration, which may improve treatment compliance. A clinical trial comparing the systemic dexamethasone exposure between the film and commercially available tablets demonstrated that the formulations were bioequivalent regarding AUC(0–t), AUC(0–∞) and Cmax [16].

The ex ante preferences for the mouth film among individuals with AARs were evaluated in a willingness-to-pay (WTP) study [17] in which 72% reported that they would prefer the mouth film over conventional corticosteroid tablets. The preferences for the oral film were manifested in a considerable WTP for the oral film that was significantly higher than the price of the tablets.

The WTP study assessed preferences from a hypothetical context where the attribute of the mouth film was described for the participants. Here, we describe the findings from a trial assessing experiences with the mouth film in a real-world clinical setting.

From a healthcare system perspective, understanding patient preferences and experiences with different alternatives is essential for informed decision-making regarding adoption, as these factors may influence treatment uptake, effectiveness in routine practice, and ultimately health outcomes.

The primary objective of the trial was to evaluate patients’ overall satisfaction with having access to the dexamethasone mouth film in relation to corticosteroid tablets in case of an AAR. Overall satisfaction includes accessibility of medication, safety/security, medication-carriage compliance, and preference of treatment over the predefined treatment period.

## 2. Methods

### 2.1. Trial Design

The trial was a Phase IV, non-randomized, open-label, single-center, low-interventional trial (trial registration: EU CT no. 2023-504975-25-00), which included patients with a moderate to severe allergy. The trial period was 6 months. The first participant was screened in January 2024, and the last participant completed in October 2024.

The investigational product (IMP) Zeqmelit^®^ 6 mg is a mouth-dissolving film containing 6 mg dexamethasone (hereafter referred to as “corticosteroid mouth film” or just “mouth film”) with a recommended dose of 1 film to be used as rescue medicine in case of an allergic reaction. The participants were instructed on how the mouth film should be administrated and informed that the different corticosteroid substances are expected to have similar efficacy in case of an AAR. The participants also had access to their standard treatment (i.e., betamethasone tablets, hereafter referred to as “corticosteroid tablets” or just “tablets”) throughout the trial. The IMP was intended to be used as an alternative to the standard treatment.

### 2.2. Study Sample

All patients with moderate to severe allergy receiving treatment at a primary care center (Kåbohälsan, Uppsala, Sweden) were assessed for eligibility based on the following criteria:

Inclusion criteria

1. Willing and able to give written informed consent for participation in the trial;
2. Generally healthy male or females ≥18 years of age;
3. Had experienced moderate to severe acute allergic reactions and were currently prescribed betamethasone tablets for treatments of such reactions.

Exclusion criteria

1. History of any clinically significant disease or disorder which, in the opinion of the Investigator, could either put the participant at risk because of participation in the trial or influence the results or the participant’s ability to take part in the trial;
2. Participant who could not handle a web-based eDiary via their own electronic device (phone, tablet);
3. Participant who was on continuous systemic corticosteroid treatment;
4. Participant who was pregnant, currently breastfeeding, or intended to become pregnant during the trial;
5. The investigator considered the participant unlikely to comply with trial procedures.

### 2.3. Study Visits and Follow-Ups

In an initial pre-screening step, potentially suitable participants were identified and invited to participate in writing. The invitation included study information and a copy of informed consent, which was to be signed before any trial-related procedures were performed.

Suitable patients were invited to a screening and inclusion visit. At the visit, they were further informed about the trial, eligibility was confirmed, and informed consent and demographics were collected. The participants were also provided with the corticosteroid mouth film, and prescriptions for corticosteroid tablets were renewed (if applicable). Moreover, the participants were introduced to an electronic diary (eDiary) and asked to provide answers to screening questions related to medical history and AAR experiences (past 12 months).

### 2.4. Monthly Follow-Ups in eDiary

The participants’ experiences with the mouth film and tablets, overall and in case of an AAR, were assessed every month (6 occasions in total) in the eDiary. The participants were reminded to log in to the eDiary every month and to complete the electronic questionnaire.

### 2.5. Ad Hoc Follow-Up in the Event of an AAR

If a participant registered an AAR in the eDiary, an alert was sent to the principal investigator. If the AAR was treated with IMP, the trial site followed up on potential adverse events (AEs) and concomitant medication for two weeks following the reporting of the AAR in the eDiary.

### 2.6. Study Assessments

#### 2.6.1. Population Characteristics

Population characteristics collected at screening and from the baseline questionnaire included demographics (age, sex, BMI), medical history (including experience of AARs in past 12 months) and medication other than corticosteroids.

#### 2.6.2. Study Outcomes

Primary and secondary outcomes were based on data collected from the eDiary monthly follow-ups (month 1 to 6). Some questions were included only in the last follow-up (month 6).

The following assessments were included in the primary outcome (overall satisfaction with mouth film versus tablets):

1. Satisfaction with accessibility of mouth film versus tablets (eDiary month 1 to 6) based on the following questions:
  (a) “How satisfied are you with how accessible your medication (the mouth film) is in the event of an AAR?” Response alternatives: very satisfied/satisfied/neither nor/a little satisfied/not at all satisfied.
  (b) “How satisfied are you with how accessible your medication (the tablets) in the event of an AAR?”. Response alternatives: very satisfied/satisfied/neither, nor/a little satisfied/not at all satisfied.
2. Feeling of safety/security with mouth film versus tablets (eDiary month 1 to 6) based on the following questions:
  (a) “Do you feel safer when you have access to the film as compared to tablets?” Response alternatives: much safer/safer/neither, nor/less safe/not safe at all.
  (b) “With access to the mouth film, have you dared to expose yourself to situations that you would have otherwise avoided?” Response alternatives: yes/no.
3. Medication-carriage compliance for mouth film versus tablets (eDiary month 1 to 6) based on the question: “Do you more often bring your medication (the mouth film) with you as compared to your prescribed medication (the tablets)?” Response alternatives: yes/no.
4. Treatment preferences, mouth film versus tablets (eDiary month 6 only) based on the question: “For your next prescription, would you prefer mouth film or tablets?” Response alternatives: mouth film/tablets/both.

The following assessments were included in the secondary outcome (user experience and perceived efficacy of the mouth film in case of an AAR):

1. Overall experience of AARs and treatment (eDiary month 1 to 6), including number of AARs during the study period, type of AARs, treatment of AARs and number of AARs requiring additional treatment (Question: “Did you need to seek acute treatment by your doctor/emergency department?” Response alternatives: yes/no.
2. AEs in relation to treatment with mouth film (2-week ad hoc follow-up), including assessment of type of AE, severity level, and whether they were unlikely, possibly or probably linked to IMP.
3. Access to mouth film in case of an AAR (eDiary month 1 to 6) based on the question, “When the allergic reaction started, did you have immediate access to the mouth film?” Response alternatives: yes/no.
4. Perceived treatment efficacy for mouth film (eDiary month 1 to 6) based on the question, “How well did the medication work?”. Response alternatives: not at all/a little/neither, nor/good/very good.

For a better understanding of treatment compliance other than for corticosteroids, the medication-carriage compliance for an EAI was also assessed based on the question, “If you have an adrenaline pen, do you always bring it with you?”. Response alternatives: yes/no/I do not have an adrenaline pen. (Note: adrenaline pen is the only available EAI in Sweden.)

### 2.7. Analysis

#### 2.7.1. Likert-Type Scales

To facilitate data interpretation and comparison between groups, qualitative responses were converted into ordinal Likert-type scale data [18] when applicable. The least positive answer alternative (i.e., “not at all”) was given the value 0, and the most positive (i.e., “very satisfied” or “much safer”) was given the value 4.

#### 2.7.2. Subgroup Analyses

Primary outcomes were analyzed for the whole study sample as well as for subgroups with and without an EAI at baseline and during the trial. We hypothesized that there could be differences in how these groups perceived the mouth film due to:

1. Potential differences in terms of AAR severity (a prescription for an EAI indicates that the individual has experienced, or is at risk of, very severe allergic reactions including anaphylaxis);
2. Different positioning of corticosteroids in the treatment regimen (less acute stage) when used in combination with an EAI.

Since previous studies have reported gender differences related to medication-carriage compliance among individuals with AARs [12], this outcome was analyzed for all participants and with stratification for gender.

In line with the trial objectives, outcomes related to satisfaction, user experience, and perceived treatment efficacy were analyzed for the subgroup of participants who used the mouth film to treat AARs during the trial period.

#### 2.7.3. Statistical Analysis

Statistical analyses were performed using Microsoft Excel, SPC for Excel (BPI Consulting LLC, Oklahoma City, OK, USA), and MedCalc Software Ltd. (Ostend, Belgium) version 23.3.4.

Descriptive statistics: Qualitative data were summarized using standard descriptive measures. For categorical variables, this included number and proportion, and for continuous variable, mean, standard deviation, and/or median and min:max. Standard errors or 95% confidence intervals (CI) were calculated for outcomes presented in figures.

Statistical comparisons: Differences in proportions between groups (e.g., % females) were assessed using a two-proportion Z-test. Differences in means between subgroups (e.g., film vs. tablets) and overall mean satisfaction ratings (e.g., Lickert estimations) for mouth film versus tablets were assessed using a Student’s *t*-test, a Mann–Whitney U-test, and a Wilcoxon signed rank test. The distribution of categorical (e.g., “not at all”, “very satisfied”) variables across subgroups was evaluated using a Pearson’s chi-square test of independence. To assess within-individual variability, we estimated the coefficient of variation (CVi) for each individual (CVi = standard deviation_i_/mean_i_ × 100) for satisfaction with accessibility and safety/security.

## 3. Results

### 3.1. Sample Overview and Participant Characteristics

A total of 50 participants were screened and enrolled in the trial, of which 44 provided answers to questions in the eDiary at any of the six-monthly follow-ups (referred to as full analysis set). Response rates for individual follow-ups were 34, 37, 34, 29 and 26 at months 1, 2, 3, 4, 5 and 6, respectively. For the inclusion flowchart, see the Supplementary Materials Figure S1.

Table 1 presents patient characteristics for the full analysis set, including subgroups with and without an EAI. Patient characteristics for the total sample (i.e., all 50 participants enrolled in the study) are provided in Supplementary Materials Table S1. More detailed information on medical history and medication at baseline for the full analysis set and total sample is provided in the Supplementary Materials Tables S2 and S3.

In the full analysis set, mean age was approximately 50 years, mean BMI was approximately 26 kg/m^2^, and the majority were female.

Most of the participants (57%) had an EAI. There were no statistical differences in terms of age, gender or BMI between subgroups with and without an EAI (Table 1).

All events reported in the medical history were related to an allergy; the most common were immune system disorders, followed by skin and subcutaneous tissue disorders and respiratory, thoracic and mediastinal disorders (Table 1). Among the immune system disorders, food allergies were the most common and reported by 37% of the participants, followed by allergies to arthropod stings, reported by 30% (see Supplementary Materials Table S2 for a full display of medical history).

Overall, 32% of the participants reported experience of one or more AARs during the 12 months prior to the screening visit, of which 79% reported experience of an AAR that required emergency treatment by their doctor or in an emergency department. These proportions were significantly higher in the subgroup without an EAI compared to the group with an EAI (Table 1).

### 3.2. Overall Satisfaction with Mouth Film Versus Tablets

#### 3.2.1. Satisfaction with Accessibility and Feeling of Safety/Security

**Figure 1:**
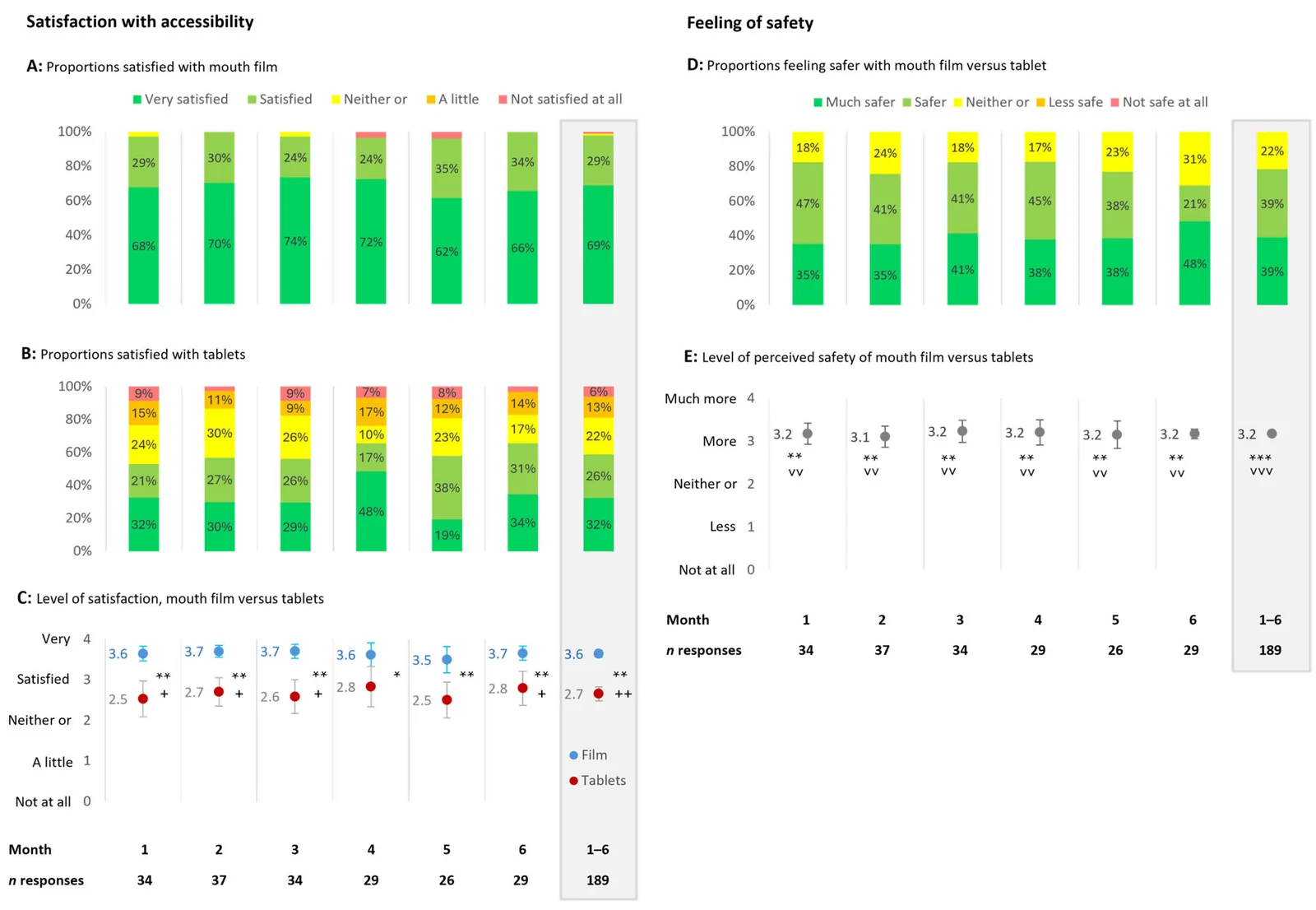
Satisfaction with accessibility and feeling of safety/security for the mouth film versus tablets over the 6-month trial period. Notes: (**A**–**C**) show participants’ satisfaction with treatment accessibility of mouth film and tablets (Question: How satisfied are you with how accessible your medication [the mouth film/the tablets] is in the event of an acute allergic reaction?). (**D**,**E**) show participants’ feeling of safety/security for mouth film versus tablets (Question: Do you feel safer when you have access to the film as compared to tablets). (**A**,**B**,**D**) show the distribution of answer alternatives for responses in eDiary at each monthly follow-up as well as for the sum of all responses collected over the 6 months (last column). (**C**,**E**) show mean Likert grades at each follow-up and for the sum of all responses over the 6 months (last column) with 95% confidence intervals. Statistically significant differences between mouth film and tablets are shown where * = *p* < 0.05, ** = *p* < 0.001, *** = *p* < 0.0001 for Student’s *t*-test; + = *p* < 0.05, ++ = *p* < 0.001 for Mann–Whitney U-test; vv = *p* < 0.001, vvv = *p* < 0.0001 for Wilcoxon signed-rank test.

A higher degree of satisfaction with accessibility was reported for the mouth film compared to tablets at each of the six follow-ups in the eDiary (Figure 1A,B). In 98% of all responses collected over the six-month period (*n* = 189), the participants reported that they were satisfied or very satisfied with accessibility to the mouth film. The corresponding figure for tablets was 58%. The proportion of responses stating that the participants were not satisfied at all were 1% for mouth film compared with 6% for tablets. Mean Likert grades showed a significantly higher degree of satisfaction with accessibility for the mouth film at every time point and based on all responses over the whole study period (Figure 1C). The estimated CVi for satisfaction with accessibility over months 1–6 (189 responses) was substantially lower for the film than for the tablets (4% vs. 19%) (*p* < 0.0001). The degree of satisfaction for the mouth film was higher among participants with an EAI compared with those without an EAI (mean Likert grades over six months: 3.72 versus 3.53, *p* < 0.01; Supplementary Materials Figure S2A).

Most participants felt safer with the mouth film compared to tablets. In 78% of responses over the six months, the participants stated that they felt safer or much safer with the mouth film, and no participants reported that they felt less safe or not safe at all. Likert grades showed that the degree of perceived safety/security varied little between the different time points, and the mean value of 3.2 for month 1–6 was statistically significant from the threshold of indifference (2.0 or “neither”) (*p* < 0.0001). The estimated CVi for safety/security was 8%. There was no statistical difference between participants with and without an EAI in terms of perceived safety/security (Supplementary Materials Figure S2B).

In 24% of all responses collected over the six-month period, the participants stated that with access to the mouth film, they would dare to expose themselves to situations that they would otherwise have avoided. Between follow-ups, this proportion ranged between 18% (month 1) and 31% (month 6).

#### 3.2.2. Medication-Carriage Accessibility Compliance

**Figure 2:**
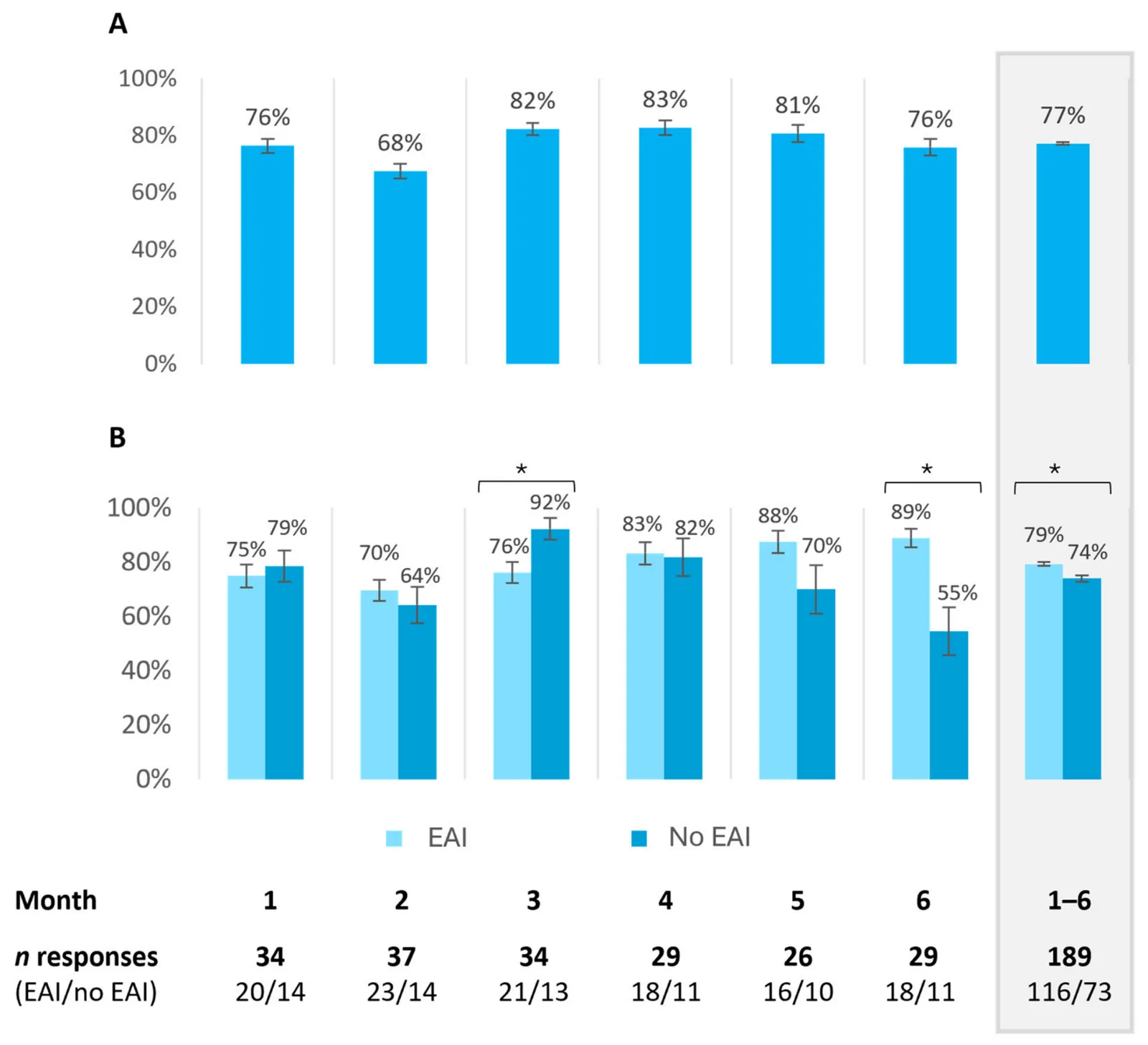
Medication-carriage compliance of mouth film versus tablets for all participants (**A**) and for participants with EAI compared with those without EAI (**B**). Abbreviations: EAI, epinephrine autoinjector. Notes: Medication-carriage compliance refers to participants’ answers to the question, “Do you more often bring your medication (the mouth film) with you as compared to your prescribed medication (the tablets)?” The figure shows proportions answering “yes” of all responses collected in the eDiary at each monthly follow-up and for the sum of all responses collected over the 6-month trial period (last column). The error-bars represent 95% confidence intervals for percentages at each follow-up. Stars indicate statistically significant differences between the subgroups with and without EAI where * = *p* < 0.05.

Most participants reported that they more often brought the mouth film with them as compared to tablets (i.e., better medication accessibility compliance with mouth film) (Figure 2A). Based on all responses over six months, the proportions reporting better medication-carriage compliance for the mouth film versus tablets was significantly higher among women than men (82% versus 66%, *p* = 0.0108).

Most participants reported that they more often brought the mouth film with them as compared to tablets (i.e., better medication-carriage compliance with mouth film) (Figure 2A). Based on all responses over six months, the proportions reporting better medication-carriage compliance for the mouth film versus tablets was significantly higher among women than men (82% versus 66%, *p* = 0.0108).

Based on all responses collected over the trial period, proportions reporting better mediation-carriage compliance for the mouth film compared to tablets were significantly higher for those with an EAI; however, this was not consistent between follow-ups (Figure 2B). Of note is that there was a larger variability in mediation-carriage compliance of mouth film versus tablets between follow-ups among participants without an EAI (range 55–92%) compared to participants with an EAI (range 70–89%) (Figure 2B).

Among participants who reported in the eDiary that they had an EAI, proportions stating that they always brought the injector with them ranged between 50% (month 1) and 28% (month 6). Among all responses over the six months, 41% stated that they always brought the injector. This proportion was significantly lower among men than women (8.3% vs. 56%, *p* < 0.0001).

#### 3.2.3. Treatments Preferences

**Figure 3:**
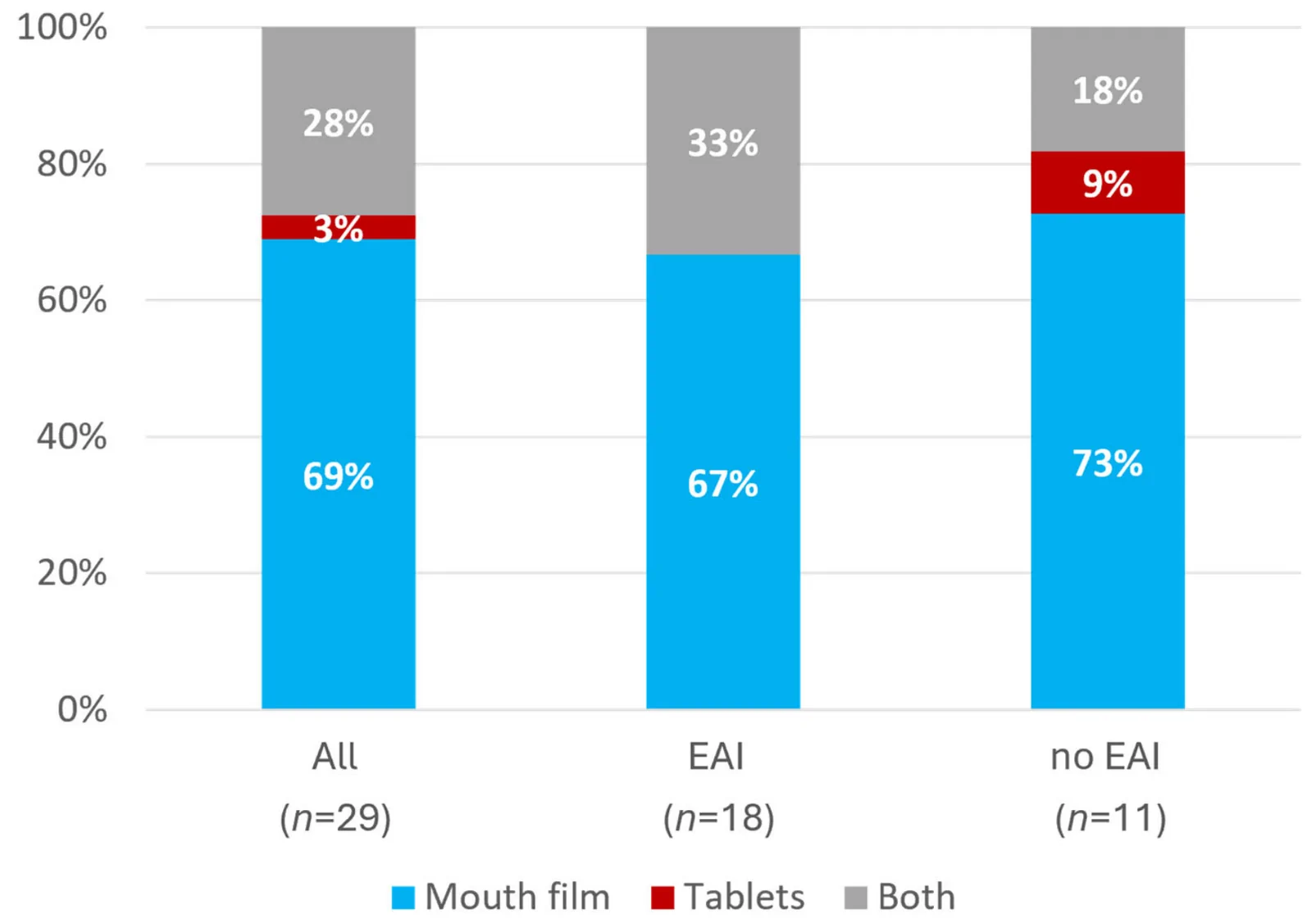
Treatment preferences at month 6. Abbreviations: EAI, epinephrine autoinjector. Notes: The figure shows proportions of participants answering mouth film, tablets, or both to the question, “ For your next prescription, would you prefer mouth film or tablets?” This question was administered at the six-month follow-up only.

Most participants who answered the eDiary questionnaire at month 6 reported that they would prefer the mouth film for their next prescription; only one individual preferred the tablets. More than a quarter preferred having both mouth film and tablets (Figure 3).

There were no statistical differences in terms of preferences for mouth film versus tablets between groups with and without an EAI.

### 3.3. User Experiences with Mouth Film in the Event of an AAR

Over the trial period, 18 AARs were reported by 13 participants in the eDiary (Table 2). Three (17%) of the AARs experienced by three participants (23%) required acute treatment by a doctor or at the emergency department.

Triggers of AARs included food allergens (four events experienced by three participants), pollen (three events by three participants), insect bites (two events by two participants) and animal fur (one event). For the remaining eight events experienced by six participants, the trigger was reported as “other”.

Eleven participants treated 16 AARs with mouth film and two participants treated two AARs with tablets (Table 2). One of the participants who chose tablets reported that the reason was that “it felt safer”, and the other participant reported that “it was the treatment I had access to”. For 33% of AARs, other medications (asthma medicine or antihistamine) were used in combination with corticosteroids. There were no reports of epinephrine being used in combination with corticosteroids to treat AARs during the trial.

For 15 of the 16 AARs (94%) treated with mouth film, the mouth film had been immediately accessible for treatment (Table 2). Moreover, for 15 of the AARs (94%), treatment efficacy was perceived as good or very good, and for 11 (69%), participants reported that the film had made them feel safe or very safe compared to tablets.

The 11 participants who used the mouth film to treat AARs during the trial showed a similar perception of safety/security of the film versus tablets compared with the remaining 32 participants in the full analysis (based on mean Likert grades for all responses collected over the six-month trial period) (Supplementary Materials Figure S3A). Satisfaction with accessibility was rated somewhat lower for both mouth film and tablets by the 11 participants who used the mouth film compared with the remaining 32 participants; however, both groups rated satisfaction with accessibility significantly higher for the mouth film compared with tablets (Supplementary Materials Figure S3B).

Among participants with AARs treated with mouth film, 11 reported a total of 56 AEs, of which 54 (96%) were assessed as unlikely related to IMP (Supplementary Materials Table S4). Two events (hypoesthesia and paraesthesia) reported by the same participant were assessed as possibly related to the mouth film. The majority (88%) of AEs were assessed as moderate in intensity, 8.9% as mild and 3.6% as severe. Most AEs were related to symptoms consistent with allergic reactions; the most common were skin and subcutaneous tissue disorders (48%) followed by respiratory, thoracic and mediastinal disorders (29%) (Supplementary Materials Table S5).

## 4. Discussion

This real-world clinical trial investigated how individuals with experience of moderate to severe AARs treated with corticosteroids perceived a dexamethasone mouth film compared to their ordinary betamethasone tablets as rescue medicine in the event of an AAR. The results show a higher degree of satisfaction with the mouth film compared to tablets, both in the study sample overall and in a subgroup of participants who gained experience from using mouth film to treat one or more AARs during the trial.

The substantially lower within-individual coefficient of variation (CVi) observed for the film compared with tablets suggests more consistent patient-reported satisfaction over time. For satisfaction with accessibility, the CVi was markedly lower for the film (4%) than for the tablets (19%), indicating considerably greater stability in perceived accessibility of the film formulation across the study period. A similarly low CVi for safety/security (8%) further supports a pattern of low intra-individual fluctuation in these perceptions. Taken together, these findings suggest that the film formulation was associated not only with higher satisfaction levels but also with more stable user experiences over time, which may be relevant for treatments where predictable patient experience and ease of use are important.

Immediate start of treatment of AARs is essential to avoid progress to a more severe condition, and this requires that the medication be easily accessible. Despite this, studies have shown a low degree of medication-carriage compliance among individuals with AARs. In a study by Andersson et al. [9], which included 387 individuals with moderate to severe allergy, 66% reported that they had experienced not having their allergy medicine immediately available when needed for an AAR; the main reason was being away from home without bringing the allergy medicine. Other studies have reported low mediation-carriage compliance of EAIs of around 50–60% [12,13] with even lower frequencies observed among men [12]. Similarly, in this trial, 50% or less of the participants with an EAI reported that they always brought their EAI with them, and the proportions were significantly lower among men. The observed differences between men and women may be attributed to gender-related behavioral patterns, particularly differences in handbag-carrying habits.

Improved medication-carriage compliance is likely to have a positive impact on treatment compliance. In the trial, nearly all participants reported that they were satisfied or very satisfied with the accessibility to the mouth film, and the degree of satisfaction was significantly higher compared to the tablets. In line with this, approximately 70–80% of the participants reported that they more often brought the mouth film with them compared to tablets. This was further supported by the high degree of medication-carriage compliance among participants using the mouth film to treat an AAR during the trial; for 15 of the 16 (~94%) AARs treated with mouth film, the participants reported having the film immediately accessible. However, it cannot be concluded whether the high medication-carriage compliance in this trial is attributable to having access to the mouth film or that participation in the trial increased the participants’ attention to corticosteroid use.

An interesting observation was that among those participants who experienced one or more AARs over the six-month trial period, only 23% required additional treatment by a physician or at an ER, compared with 79% among those with AAR(s) in the year before the trial. This could be a consequence of better accessibility to corticosteroid treatment in the trial, which may have resulted in improved treatment compliance and less likelihood for progress to a more severe allergic reaction. In line with this, a vast majority reported good or very good effect of the mouth film. Although the trial was not primarily designed to evaluate efficacy of the mouth film, the participants were overall satisfied with the treatment effect. Another potential explanation for less interaction with the healthcare system in connection with AARs is an improved feeling of safety/security.

Previous studies have indicated that AARs can contribute to anxiety and restriction of social life and daily activities with a negative impact on quality of life [8,9,10,11]. For example, in Andersson et al. [9], most of the participants reported that they were worried about having an allergic reaction. Moreover, 50% of the participants reported that their allergy prevented them from participating in leisure activities, and 85% reported that they had experienced AARs that had interrupted daily activities. The participants in this trial reported an improved feeling of safety/security with the mouth film compared to tablets, both overall and in the event of an allergic reaction. In addition, about one quarter reported that they dared to a greater extent to expose themselves to new situations with the mouth film compared with tablets. This indicates that the improved feeling of security affected their behaviors and positively influenced their daily life. Consequently, the study participants showed a strong preference for the mouth film over tablets. The majority reported that they would prefer to have the mouth film for their next prescription, and 85% of participants who experienced one or more AARs during the trial chose to use the mouth film instead of tablets.

Subgroup analyses were performed to investigate if the experiences and perceptions of the mouth film versus tablets differed between participants with and without an EAI. We hypothesized that a subgroup with an EAI would represent more severe AARs, including anaphylaxis. We further hypothesized that the less acute positioning of corticosteroids in the treatment regimen when used with an EAI could affect how the mouth film was perceived and valued. Unexpectedly, the group with an EAI reported fewer AARs in the year prior to baseline, including those that had required treatment by a general practitioner or in the ER. Intuitively, this could be interpreted as if the group with the EAI had less severe allergies and AARs. However, it may instead reflect that individuals at risk of reactions with anaphylaxis are more cautious in avoiding exposure to allergens. Another potential reason is undertreatment in the group without an EAI. The only difference between subgroups in terms of experiences of the mouth film versus tablets was that the group with an EAI showed a somewhat higher satisfaction with accessibility.

In terms of safety, the results from ad hoc follow-ups of participants using the mouth film to treat ARRs indicated that the film was well tolerated, and no safety concerns were identified in the trial.

Designing clinical trials for AARs is challenging due to the unpredictable and variable nature of allergic reactions. The trial design, which was chosen to gain real-life experience with the mouth film, has inherited strengths and limitations. The trial was non-randomized, and the corticosteroid for treatment of AARs was administrated by open label. Importantly, the participants had access to both the mouth film and tablets, which allows capturing individual preferences for the treatment options. Although one might expect individuals in an acute situation to choose a treatment with which they are already familiar, this study showed the opposite: the majority (~85%) of those who had experience with an AAR used the mouth film rather than the tablets. The finding is consistent with a previous preference study, in which 74% of individuals without prior experience of the mouth film nevertheless preferred it over tablets [17]. This somewhat alleviates concerns that the questions in the eDiary may have been overly tailored to this specific trial, as no validated questionnaire addressing the study objectives was available. At enrollment, all participants had previously been prescribed corticosteroid tablets for use in the event of an AAR. Consequently, some questions referred to their prior experience with this medication to enable comparisons with the mouth film. It therefore seems unlikely that the framing of the questionnaire introduced any substantial bias into the eDiary responses.

To mimic a real-life situation, the trial was low-interventional, with no regular physical visits and infrequent completion of the eDiary. This could introduce a recall bias, but it is unlikely that it would include AARs needing treatment with a corticosteroid. However, other responses in the eDiary should be regarded as point estimates at the time for data entry. Another limitation of the trial is that participants were recruited from only one primary care center, which could influence the generalizability of the results.

Another limitation of the trial is that participants were recruited from a single primary care center located in a university town, which may limit the generalizability of the findings. The study population may differ from the broader patient population with respect to sociodemographic characteristics, educational level, health literacy, and attitudes towards novel treatments. Consequently, the findings may not be fully representative of patients treated in other primary care settings.

The requirement for participants to use a web-based eDiary via their own electronic device may have introduced selection bias by excluding individuals with lower digital literacy, limited access to digital technologies, or reduced ability to use such tools. Previous research has shown that digital health literacy varies across demographic and socioeconomic groups and may influence engagement with digital health interventions and health decision-making [19,20,21]. Consequently, the study population may have been more health-literate and receptive to novel technologies than the broader population, potentially limiting the generalizability of the preference findings.

A further limitation is that health-related quality of life (HRQoL) was not assessed in the trial. Although generic health-related quality of life instruments, such as the EQ-5D, may not be sensitive enough to directly capture the QoL loss associated with an acute event [22], the EQ-5D could potentially be sensitive enough to detect the decrease in QoL resulting from the perceived risk of experiencing an AAR as a consequence of anxiety. Since anxiety is a key component of allergic reactions, it would be advantageous for future studies to include a generic instrument to better capture a broader definition of QoL and to examine how it evolves over time following the introduction of a new treatment.

A strength of the trial is that the participants had a history of moderate to severe allergy with experience of AARs needing treatment with corticosteroids, and more than half had an EAI, thus being assessed by their treating physician to be at risk developing a severe/life-threatening AAR.

The study included patients with a diagnosis of allergy set by a physician, for which they had been prescribed corticosteroid. It reflects the perspective of patients in the primary health care center, where patients often have unclear or diffuse allergic symptoms that impact their everyday life. These patients often suffer a high degree of worry with regards to when, where and how they may suffer AARs.

In conclusion, the participants in this real-world clinical trial showed an overall preference for a corticosteroid mouth film over conventional tablets for treatment of AARs. The results indicate that the mouth film provides better medication-carriage compliance and may also enhance daily quality of life through a greater sense of security.

## Figures and Tables

**Table 1:** Participant demographics and medical history at baseline.

	Total, *n* = 44	EAI, *n* = 25	No EAI, *n* = 19
Age (years), mean (SD)	49.7 (15.5)	49.4 (15.2)	50.1 (16.2)
Gender, females *n* (%)	33 (75%)	19 (76%)	14 (74%)
BMI (kg/m^2^), mean (SD)	26.3 (4.24)	26.6 (4.77)	26.0 (3.36)
**Medical history, *n* (%) participants with events**			
Immune system disorders	38 (86%)	24 (96%)	15 (79%)
Respiratory, thoracic and mediastinal disorders	6 (14%)	2 (8%)	4 (21%) **
Skin and subcutaneous tissue disorder	8 (18%)	3 (12%)	5 (26%) **
**Medication at baseline and throughout the trial, *n* (%)**			
Oral corticosteroids	44 (100%)	25 (100%)	19 (100%)
Epinephrine	25 (57%)	25 (100%)	0 (0%)
Antihistamines for systemic use	29 (66%)	17 (68%)	12 (63%)
Asthma medication, inhaled	18 (41%)	11 (44%)	7 (37%)
Topical nasal or ophthalmic medication	3 (7.0%)	1 (4.0%)	2 (11%)
**Experience of an AAR past 12 months, *n* (%)**	**14/44 (32%)**	**5/25 (20%)**	9/19 (47%) **
1	2/44 (5%)	0/25 (0%)	2/19 (11%)
2–3	5/44 (11%)	3/25 (12%)	2/19 (11%)
>3	7/44 (16%)	2/25 (8%)	5/19 (26%) **
Required oral corticosteroids	14/44 (32%)	5/25 (20%)	9/19 (47%) **
Required acute treatment by doctor or in the emergency department	11/44 (25%)	5/25 (20%)	6/19 (32%) **

Abbreviations: AARs, acute allergic reactions; BMI, body mass index; EAI, epinephrine autoinjector; SD, standard deviation. Notes: Stars indicate statistically significant differences (two-proportion Z-test) between the subgroups with and without epinephrine autoinjector where ** = *p* < 0.001.

**Table 2:** Experiences of AARs and treatment over the trial period.

AAR Experiences over the Trial Period	Events, *n* = 18	Participants with Event, *n* = 13
An AAR required acute treatment by general physician or at the emergency department ^a^, *n* (%)	3/18 (17%)	3/13 (23%)
Treatment of ARR, *n* (%)		
Corticosteroid mouth film	16/18 (89%)	11/13 (85%)
Corticosteroid tablets	2/18 (11%)	2/13 (15%)
Other rescue medication (antihistamine or asthma medicine)	6/18 (33%)	6/13 (46%)
**Experience of AARs treated with mouth film**	**Events,** ***n*** **= 16**	**Participants with event, *n* = 11**
Immediate access to mouth film ^b^, *n* (%)	15/16 (94%)	10/11 (91%)
Perceived efficacy of mouth film ^c^, *n* (%)		
A little	1/16 (6.3%)	1/11 (9.1%)
Good	10/16 (63%)	8/11 (73%)
Very good	5/16 (31%)	4/11 (36%)
Feeling of safety/security with mouth film compared to tablets ^d^, *n* (%)		
Less safe	1/16 (6.3%)	1/11 (9.1%)
Neither, nor	4/16 (25%)	3/11 (27%)
Safe	8/16 (50%)	6/11 (55%)
Very safe	3/16 (19%)	2/11 (18%)

Abbreviations: AARs, acute allergic reactions. Notes: ^a^ Based on question: “Did you need to seek acute treatment by your doctor/emergency department?” ^b^ Refers to participants answering yes to the question, “When the allergic reaction started, did you have immediate access to the mouth film?” ^c^ Based on question, “How well did the medication work?” ^d^ Based on the question, “Did the film make you feel calmer and safer in connection to the allergic reaction as compared to tablet treatment?”.

## Data Availability

Data not provided in the main manuscript or in Supplementary Materials can be provided upon reasonable request.

## Supplementary Materials

The following supporting information can be downloaded at: https://www.mdpi.com/article/10.3390/jmahp14030043/s1, Figure S1: Study overview and inclusion flowchart; Figure S2: Satisfaction with accessibility for mouth film and tablets (A) and perceived safety for mouth film versus tablets (B)—comparison between subgroups with and without EAI; Figure S3: Satisfaction with accessibility for mouth film and tablets (A) and perceived safety for mouth film versus tablets (B)—comparison between subgroups with and without experience of using mouth film to treat AAR treated during the trial period; Table S1: Patient characteristics for all participants enrolled in study; Table S2: Medical history by system organ class and preferred term for the full analysis set (i.e., the 43 participants with answers in eDiary) and total sample (i.e., the 50 participants enrolled in the study); Table S3: Medication at baseline and throughout the trial for the full analysis set (i.e., the 43 participants with answers in eDiary) and total sample (i.e., the 50 participants enrolled in the study); Table S4: Overview of adverse events among the 11 participants who reported one or more AARs treated with mouth film in the eDiary; Table S5: Adverse events by system organ class and preferred term among the 11 participants who reported one or more AARs treated with mouth film in the eDiary.
