# Supplementary material for: A Real-World Clinical Trial Evaluating Satisfaction and Experiences with a Dexamethasone Mouth Film Compared to Corticosteroid Tablets as Rescue Medication for Moderate to Severe Acute Allergic Reactions
Source: J Mark Access Health Policy. 2026 Jul 27;14(3):43. doi: 10.3390/jmahp14030043 (PMC13511584; doi:10.3390/jmahp14030043)
Supplement: Supplementary file 1 [file jmahp-14-00043-s001.zip › jmahp-4377325-supplementary.pdf]

Bjermer et al. Supplementary Material

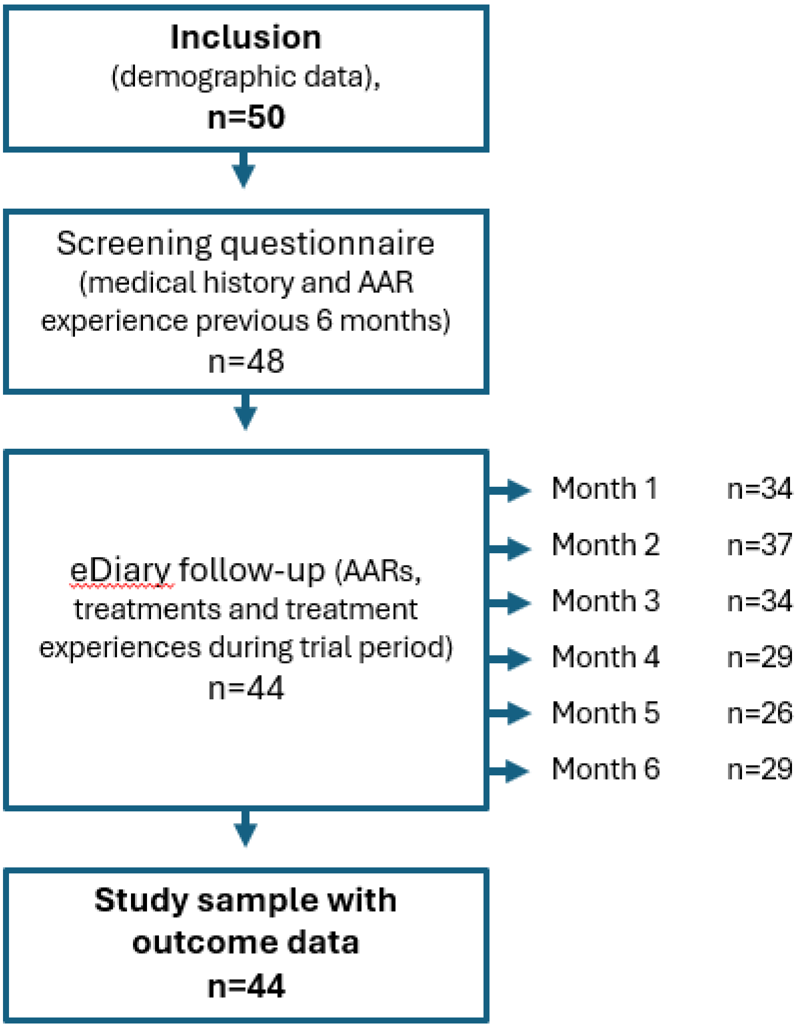

Supplementary Figure S1. Study overview and inclusion flowchart.
Abbreviations: AAR, acute allergic reaction

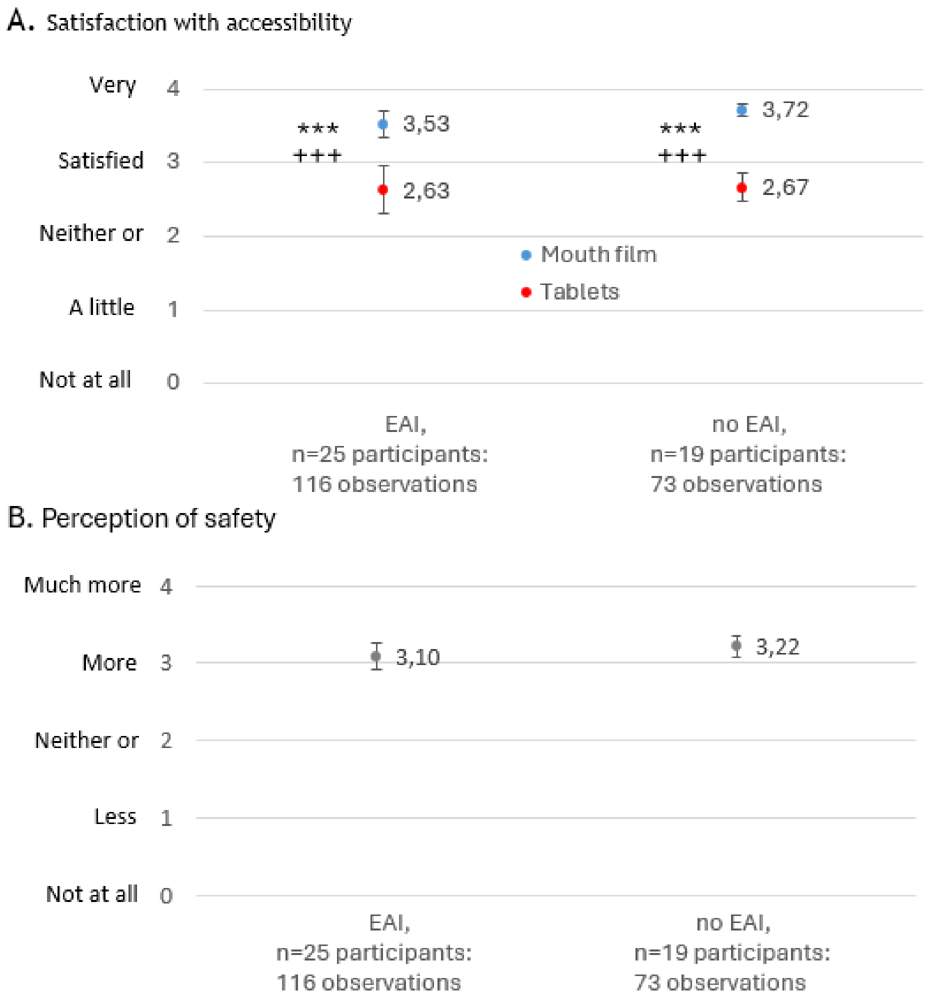

Supplementary Figure S2. Satisfaction with accessibility for mouth film and tablets (A) and perceived safety for mouth film versus tablets (B) - comparison between subgroups with and without EAI.

*Notes: The figure presents mean Likert grades with 95% confidence intervals based on all responses collected from the eDiary over the 6-month trial period. A shows participants' satisfaction with treatment accessibility of mouth film and tablets (Question: How satisfied are you with how accessible your medication [the mouth film/the tablets] is in the event of an acute allergic reaction?). B shows participants' feeling of safety for mouth film versus tablets (Question: Do you feel safer when you have access to the film as compared to tablets). ***=p<0.0001 (Student's t-test), +++=p<0,0001 (Mann-Whitney's U-test). EAI: epinephrine autoinjector*

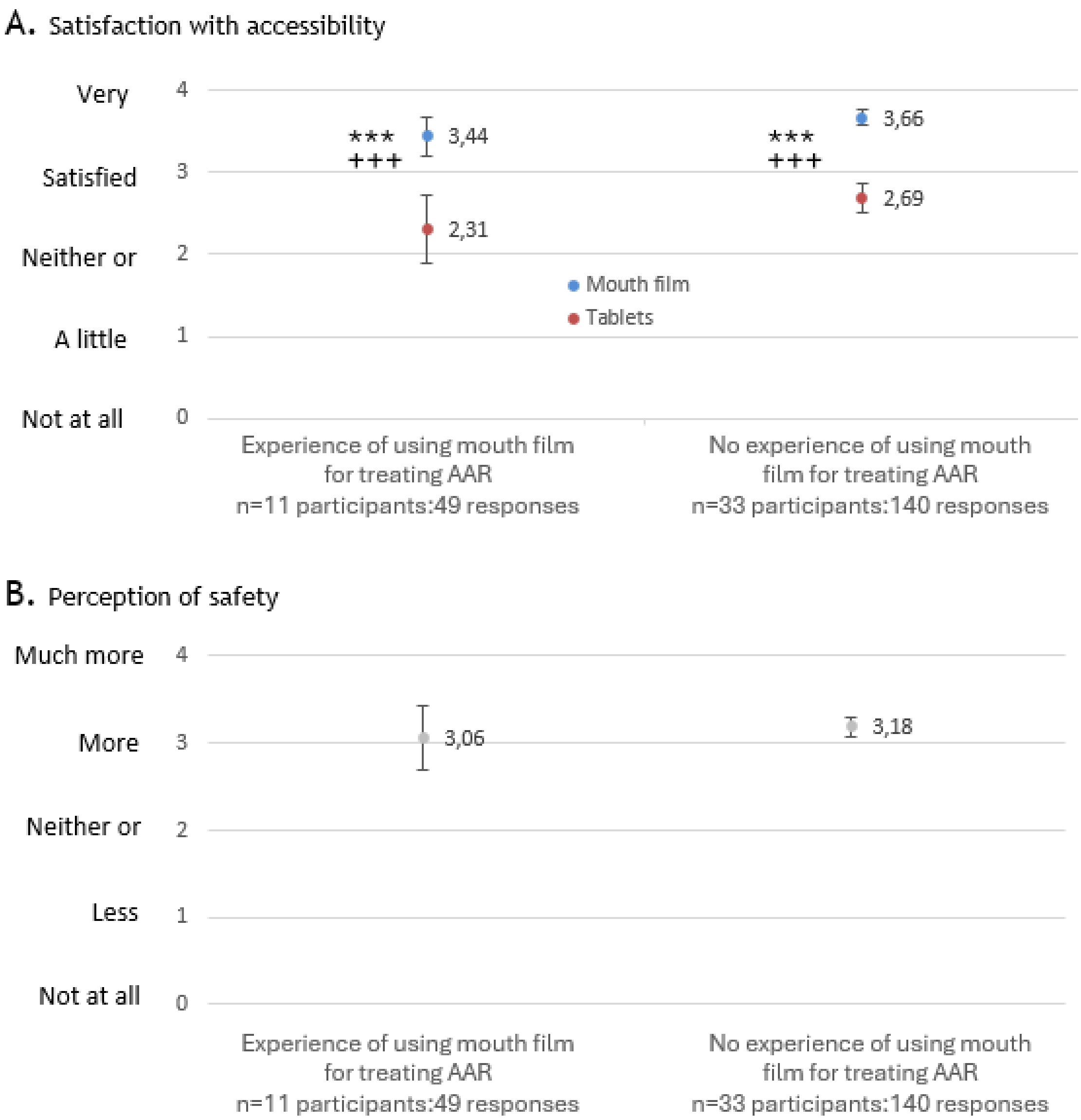

Supplementary Figure S3. Satisfaction with accessibility for mouth film and tablets (A) and perceived safety for mouth film versus tablets (B) - comparison between subgroups with and without experience of using mouth film to treat AAR treated during the trial period.

*Notes: The figure presents mean Likert grades with 95% confidence intervals based on all responses collected from the eDiary over the 6-month trial period. A shows participants' satisfaction with treatment accessibility of mouth film and tablets (Question: How satisfied are you with how accessible your medication [the mouth film/the tablets] is in the event of an acute allergic reaction?). B show participants' feeling of safety for mouth film versus tablets (Question: Do you feel safer when you have access to the film as compared to tablets). ***=p<0.0001 (Student's t-test), +++=p<0,0001 (Mann-Whitney's U-test). AAR: acute allergic reaction.*

Supplementary Table S1. Patient characteristics for all participants enrolled in study

	Total sample<br>N=50
Age *(years), mean (SD)*	48.5 (15.8)
Sex, females n (%)	34 (68%)
BMI (kg/m^2), mean SD	26.5 (4.48)
Medical history, n (%) participants with events	
Immune system disorders	45 (90%)
Respiratory, thoracic and mediastinal disorders	7 (14%)
Skin and subcutaneous tissue disorders	5 (10%)
Medication at baseline and throughout the trial, n (%)	
Oral glucocorticoids	50 (100%)
Epinephrine	29 (58%)
Antihistamines for systemic use	37 (74%)
Asthma medication, inhaled	24 (48%)
Topical nasal or ophthalmic medication	11 (22%)
Experience of AAR past 12 months[a], n(%)	15 (31%)
0-1, n (% of AARs)	3 (20%)
2-3, n (% of AARs)	5 (33%)
>3, n (% of AARs)	7 (47%)
Required oral corticosteroids, n (% of AARs)	15 (100%)
Required acute treatment by doctor or in ER, n (% of AARs)	11 (73%)

Abbreviations: AAR, acute allergic reactions; BMI, body mass index; SD, standard deviation [a]Data available for 48 participants

Supplementary Table S2. Medical history by system organ class and preferred term for the full analysis set (i.e. the 43 participants with answers in eDiary) and total sample (i.e. the 50 participants enrolled in the study).

	Full analysis set (N=44)		Total sample (N=50)	
System organ class<br>Preferred term	n participants (%)	n events	n participants (%)	n events
Total	44 (100%)	73	50 (100%)	84
Immune system disorders	38 (86%)	60	45 (90%)	69
Allergy to animal	0 (0%)	0	1 (2.0%)	2
Allergy to arthropod sting	13 (30%)	13	13 (26%)	13
Drug hypersensitivity	3 (6.8%)	5	3 (6.0%)	5
Food allergy	16 (36%)	25	21 (42%)	33
Hypersensitivity	8 (18%)	8	9 (18%)	9
Seasonal allergy	6 (14%)	7	6 (12%)	7
Respiratory, thoracic and mediastinal disorders	5 (11%)	6	7 (14%)	8
Asthma	4 (9.1%)	4	6 (12%)	6
Rhinitis	2 (4.5%)	2	2 (4.0%)	2
Skin and subcutaneous tissue disorders	4 (9.1%)	6	5 (10%)	7
Angioedema	3 (6.8%)	3	4 (8.0%)	4
Idiopathic urticaria	1 (2.2%)	1	1 (2.0%)	1
Urticaria	2 (4.5%)	2	2 (4.0%)	2

Supplementary Table S3. Medication at baseline and throughout the trial for the full analysis set (i.e. the 43 participants with answers in eDiary) and total sample (i.e. the 50 participants enrolled in the study).

**Medication**	Full analysis set, N=44	Total sample, N=50
**Oral corticosteroids**	**44**	**50**
betamethasone	44	*50*
prednisolone	1	*1*
**Epinephrine**	**25**	**29**
**Antihistamines, systemic**	**30**	**37**
cetirizine	2	*4*
clemastine	1	*1*
desloratadine	25	*30*
fexofenadine	1	*1*
loratadine	2	*2*
**Asthma medication, inhaled**	**23**	**24**
SABA		
salbutamol	5	*8*
terbutaline	8	*8*
ICS/LABA		
formoterol and budesonide	11	*12*
formoterol and beclometasone	4	*4*
ICS		
budesonide	3	*3*
fluticasone	1	*1*
ciclesonide	0	*2*
**Topical nasal medication**	**11**	**11**
CS		
fluticasone	2	*1*
mometasone	5	*5*
CS/antihistamine		
fluticasone/azelastine	5	*5*
mometasone/olopatadine	1	*1*
Antihistamine		
levocabastine	3	*1*
**Topical ophthalmic medication**	**1**	**2**
Antihistamine		
levocabastine	1	*2*
**Other medication**	**4**	**4**
paracetamol	1	*1*
lamotrigine	1	*1*
montelukast	2	*2*
grass pollen extract	1	*1*

Supplementary Table S4. Overview of adverse events among the 11 participants who reported one or more AARs treated with mouth film in the eDiary

	n (%) participants	n events
**Any adverse event**	11 (100%)	56
**Any serious adverse event**	0	0
**Any adverse event leading to withdrawal from study**	0	0
**Any adverse event leading to death**	0	0
**Causality to mouth film**		
Possibly Related	1 (9.0%)	2
Unlikely Related	11 (100%)	54
**Severity**		
Mild	4 (36%)	5
Moderate	9 (82%)	49
Severe	1 (9.0%)	2

Supplementary Table S5. Adverse events by system organ class and preferred term among the 11 participants who reported one or more AARs treated with mouth film in the eDiary

System organ class<br>Preferred term	n (%) participants	n events
**Any adverse event**	**11 (100%)**	**56**
**Skin and subcutaneous tissue disorders**	**8 (73%)**	**27**
Angioedema	6 (55%)	12
Urticaria	5 (45%)	15
**Respiratory, thoracic and mediastinal disorders**	**6 (55%)**	**16**
Asthma	3 (27%)	5
Dysphonia	1 (9.0%)	3
Dyspnoea	3 (27%)	3
Nasal congestion	1 (9.0%)	1
Rhinitis	1 (9.0%)	1
Sneezing	1 (9.0%)	3
**Eye disorders**	**1 (9.0%)**	**5**
Lacrimation increased	1 (9.0%)	5
**General disorders and administration site conditions**	**1 (9.0%)**	**1**
Swelling	1 (9.0%)	1
**Nervous system disorders**	**2 (18.0%)**	**3**
Dizziness	1 (9.0%)	1
Hypoaesthesia	1 (9.0%)	1
Paraesthesia	1 (9.0%)	1
**Gastrointestinal disorders**	**1 (9.0%)**	**1**
Nausea	1 (9.0%)	1
**Investigations**	**1 (9.0%)**	**3**
Heart rate increased	1 (9.0%)	3
